# Genetic Architecture of Cerebral White Matter Hyperintensities in Diverse Hispanic/Latino Adults

**DOI:** 10.1212/NXG.0000000000200305

**Published:** 2025-10-02

**Authors:** Myriam Fornage, Rui Xia, Adriana Ordonez, Tamar Sofer, Carmen R. Isasi, Richard B. Lipton, Ariana M. Stickel, Wassim Tarraf, Hector M. Gonzalez, Charles S. Decarli

**Affiliations:** 1 Brown Foundation Institute of Molecular Medicine McGovern Medical School, The University of Texas Health Science Center at Houston;; 2 Human Genetics Center School of Public Health, The University of Texas Health Science Center at Houston;; 3 Department of Medicine, Brigham and Women's Hospital, Boston, MA;; 4 Department of Biostatistics, Harvard T.H. Chan School of Public Health, Boston, MA;; 5 CardioVascular Institute, Beth Israel Deaconess Medical Center, Boston, MA;; 6 Department of Epidemiology and Population Health, Albert Einstein College of Medicine, Bronx, NY;; 7 Department of Neurology, Albert Einstein College of Medicine, Bronx, NY;; 8 Department of Psychology, San Diego State University, CA;; 9 Institute of Gerontology and Department of Healthcare Sciences, Wayne State University, Detroit, MI;; 10 Department of Neurosciences, University of California San Diego, La Jolla; and; 11 Department of Neurology, University of California Davis, Sacramento.

## Abstract

**Background and Objectives:**

Cerebral white matter hyperintensities (WMHs) on MRI are part of the spectrum of age-related brain vascular injury and are associated with increased risk of stroke and dementia. Genome-wide association studies (GWASs) conducted mostly in populations of European ancestry have identified several genetic loci. Although Hispanic/Latino adults have a greater burden of WMHs than their non-Hispanic White counterparts, they are vastly underrepresented in genetic studies. We sought to characterize the genetic architecture of WMHs in a Hispanic/Latino cohort by investigating the transferability of known WMH genetic loci and by leveraging Hispanic/Latino genetic diversity to map novel loci.

**Methods:**

We conducted genome-wide association and admixture mapping analyses of WMH volume in a sample of 2,159 diverse Hispanic/Latino adults (mean age: 62.4 years; 66% female). We investigated associations at 27 previously identified WMH loci. To identify additional loci, we meta-analyzed our genome-wide association results with those of the largest GWASs published to date.

**Results:**

Accounting for population differences in linkage disequilibrium, we found some evidence of transferability of 20 of the 27 known WMH loci. Owing to power limitations, we could not exclude transferability of the remaining loci. Multiancestry meta-analysis combining our Hispanic/Latino genome-wide association results with those from a GWAS of non-Hispanic White (NHW) and African American (AA) populations identified a novel locus on 12q22 (*p* = 1.8 × 10^−8^) near *NTN4* and tagged by rs10859915, which was previously associated with blood pressure and is an expression quantitative trait locus of *AMDHD1*. Admixture mapping identified a novel locus on 14q13.2, where higher counts of European ancestry at that locus were significantly associated with higher WMH volume (*p* = 4.9 x 10^−7^). This locus spans an 800-kilobase region containing *RALGAPA1,* with known impact on neuronal function and brain development. Aggregated rare coding variants in this gene were associated with WMHs in a previous analysis of 20,719 stroke-free and dementia-free adults.

**Discussion:**

Our study suggests that WMH loci previously identified in NHW and AA individuals are relevant to Hispanic/Latino adults. It demonstrates the power of the diverse Hispanic/Latino population to fine-map known genetic loci and discover novel ones, augmenting our understanding of the genetic architecture of cerebral WMHs.

## Introduction

Cerebral white matter hyperintensities (WMHs) on MRI are defined as areas of signal hyperintensity in the deep or periventricular white matter on brain MRI T2-weighted or fluid-attenuated inversion recovery (FLAIR) sequences.^1^ WMHs are part of the spectrum of brain vascular injury that accompanies aging and reflects cerebral small vessel disease.^2^ WMHs influence brain and cognitive health as early as midlife^3,4^ and, in later life, are associated with stroke, dementia, and death.^5^ In addition to age, vascular risk factors, most notably, hypertension, play a major role in WMH etiology.^6^ Moreover, WMHs are highly heritable, with heritability estimates ranging from 55% to 80%.^7-9^ To date, genome-wide association studies (GWASs) have identified 27 loci associated with WMH burden.^10-13^ These genetic discoveries have implicated genes related to the structure and function of the extracellular matrix and have further underscored the role of hypertension as a major risk factor of WMHs, with approximately half of the identified loci also associated with blood pressure levels. However, the loci identified to date explain only a fraction (<10%) of the WMH SNP-based heritability, estimated at 29%.^13^ Furthermore, they were discovered predominantly in populations of European ancestry.^13^ This bias impedes our ability to fully understand the genetic architecture of WMHs and has important implications for future applications of genomic medicine across global populations, possibly exacerbating health disparities.^14,15^

Hispanic/Latino individuals comprise the largest ethnic or racial minority group in the United States, with an estimate of 65.2 million, representing 19.5% of the overall US population in 2023.^16^ Racial and ethnic differences in WMH prevalence and severity haven been documented, with Hispanic/Latino and African American (AA) adults having a greater burden of WMHs than non-Hispanic White (NHW) adults, possibly due to differences in vascular risk factor burden.^17^ The genetic architecture of WMHs in Hispanic/Latino populations remains largely unexplored, with only 1 study of Caribbean Hispanic adults published to date that reported limited findings and had limited generalizability to other Hispanic/Latino populations.^18^

We leveraged the diverse sample of Hispanic/Latino adults from the Hispanic Community Health Study/Study of Latinos (HCHS/SOL) to investigate the genetic architecture of WMHs using the complementary approaches of GWASs and admixture mapping.

## Methods

### Study Participants

Participants were selected from the Study of Latinos Investigation of Neurocognitive Aging (SOL-INCA), an ancillary study of the HCHS/SOL, which prospectively assesses the cognitive performance of adults aged 50 years and older. The design, cohort selection, and recruitment procedures for HCHS/SOL and SOL-INCA have been previously described.^19-21^ In brief, at HCHS/SOL visit 1 (2008–2011), 16,415 Hispanic/Latino adults (aged 18–74) were enrolled from diverse communities in Bronx, NY; Chicago, IL; Miami, FL; and San Diego, CA. At visit 1, participants older than 45 years underwent a cognitive assessment. A subsample of those aged 50 or older at visit 1 were reassessed at visit 2 (2015–2018) as part of SOL-INCA (N = 6,377). The Study of Latino–Investigation of Neurocognitive Aging–MRI (SOL‐INCA‐MRI) substudy was designed to investigate brain health and aging using advanced MRI techniques. Brain MRI data collection targeted a total of 2,668 participants, including 2,323 participants recruited from SOL-INCA, with participant selection oversampling individuals with cognitive impairment and the remaining cognitively healthy participants randomly sampled with sex and field center matching to the participants with cognitive impairment. In addition, 272 participants aged between 35 and 50 years at visit 2 were randomly selected from the HCHS/SOL parent cohort to broaden the age range and provide a lifespan perspective on Hispanic/Latino brain health.

### Standard Protocol Approvals, Registrations, and Participant Consents

The study was approved by the institutional review boards at each of the participating institutions. All participants provided written informed consent.

### MRI Methodology and WMH Assessment

MRI scans were obtained using 3T scanners and interpreted using a standardized protocol developed at the IDeA Laboratory at the University of California Davis. All images were quality checked visually at every step of the segmentation process. Skull stripping, or removal of nonbrain tissue, was performed using a convolutional neural network model.^22^ WMH volume was obtained using a modified Bayesian probability structure–based algorithm on FLAIR and 3D T1 images.^23^ Details about the method and its reliability have been previously described.^24,25^ WMH volume measures were log-transformed to reduce skewness. In addition, we generated inverse normal–transformed values following the same analytical plan as that of the previously published GWASs^13^ to allow for comparisons and meta-analyses.

### Genotypes and Imputation

Details of genotyping and quality control procedures were reported elsewhere.^26^ In brief, 12,874 participants provided consent and a DNA sample for array genotyping using an Illumina custom array, SOL HCHS Custom 15041502 B3, consisting of the Illumina Omni 2.5M array (HumanOmni2.5-8v1-1) and approximately 150,000 custom single-nucleotide polymorphisms (SNPs). Quality control procedures^27^ excluded 71 participants with gender mismatch, chromosome abnormalities, or a missing call rate >1%. An additional 19 individuals with significant Asian ancestry were also excluded, resulting in a total of 12,774 samples successfully genotyped for 2,232,944 SNPs. These genotypes were then prephased and imputed with the 1,000 Genomes (Phase 3) and TOPMed (freeze 5b) reference panels.^28^ Genotype and brain MRI data were available for 2,159 individuals.

### Genetic Analysis Groups

HCHS/SOL participants self-identified as primarily belonging to one of 6 background groups: Central American, Cuban, Dominican, Mexican, Puerto Rican, and South American. Based on these groups, a “genetic analysis group” variable was constructed using a multidimensional clustering method.^26^ The genetic analysis groups are similar to self-identified background groups regarding cultural and environmental characteristics but are more genetically homogeneous. From these, we defined a Mainland group that included individuals belonging to the Mexican, Central American, and South American genetic analysis groups and a Caribbean group that included individuals belonging to the Cuban, Dominican, and Puerto Rican genetic analysis groups. The Mainland group includes groups with higher proportions of Amerindian ancestry, with the Mexican subgroup generally having the highest, while the Caribbean group includes groups with higher proportions of African ancestry, with the Dominican subgroup generally having the highest. Additional information about the distribution of admixture proportions in HCHS/SOL has been previously reported.^26^

### Estimation of WMH Heritability Tagged by Common Genetic Variants

We estimated WMH SNP-based heritability via the Haseman-Elston method-of-moment estimator^29^ using the variance explained by the kinship matrix, representing the additive effects of common genetic variants.^30^ Estimations were performed on all individuals and also excluding first-degree and second-degree relationships based on the kinship coefficients.

### Genome-Wide Association Analyses and Meta-Analyses

Genome-wide association analyses were performed using a linear mixed model implemented in the GENetic EStimation and Inference in Structured sample (GENESIS) Bioconductor package.^31^ The model was specified to allow for heterogeneous residual variances among ancestry groups defined by the “genetic analysis group.” Correlations between individuals were modeled via kinship, household, and census block–unit sharing matrices. All analyses were adjusted for age at MRI, sex, total intracranial volume, MRI scanner, and 5 principal components of ancestry. GWAS analyses were performed in the total sample and in the Mainland and Caribbean subgroups. Primary analyses were performed on the WMH inverse normal–transformed values and secondary analyses on the log-transformed values. In addition, gene-based association tests were conducted using the Multi-marker Analysis of GenoMic Annotation (MAGMA),^32^ with *p* < 2.6 × 10^−6^ as a gene-wide significance threshold.

We also combined our GWAS results with the previously published GWAS summary results from European and African ancestry populations^13^ using 2 methods: (1) a fixed-effects inverse-variance weighted meta-analysis using METAL^33^ and (2) a multiancestry meta-regression implemented in the Meta-Regression of Multi-AncEstry (MR-MEGA), which partitions allelic effect heterogeneity into components because of population background and residual variation.^34^ In all GWAS analyses and meta-analyses, the threshold of *p* < 5 × 10^−8^ was used to identify genome-wide significant variants.

### Assessment of Transferability of GWAS Loci

At each of the 27 previously known loci, we generated a credible set of independent variants consisting of the lead SNP and its proxies located within a 50-kb window and in linkage disequilibrium (LD) (*r*^2^ ≥ 0.6) based on the European ancestry 1,000 Genomes data (except for 10p14, which was based on the African ancestry 1,000 Genomes data because this locus was identified in a GWAS of African ancestry population). Proxies were required to have *p* < 5 × 10^−5^ in the original GWAS. A locus was deemed transferable if at least 1 variant in the credible set was associated with *p* < 0.05 and matching direction of effect in our data set. To account for the multiple tests performed, we also calculated FDR-adjusted *p* values (Q values) of association, which are provided for information.

Because low statistical power may hinder our ability to draw appropriate conclusions regarding a locus' transferability, we calculated the statistical power to observe an association at the lead variant in each locus using the effect size estimate from the published GWAS, the allele frequency of the variant, and the sample size in our data set, assuming alpha = 0.05.

### Trans-Ancestry Colocalization

We implemented TAColoc^35^ for trans-ancestry colocalization analysis. The method uses the joint likelihood mapping statistic,^36^ which accounts for LD structure, to estimate the posterior probabilities of colocalization between GWAS signals, and compares them with the probabilities of distinct causal variants. Analyses were performed within a 50-kb window for each known WMH locus. LD in European and African ancestry populations was estimated from the 1,000 Genomes data. LD for HCHS/SOL Hispanic participants was estimated directly from the genotype data.

### Local Ancestry Estimation and Admixture Mapping Analysis

Local ancestry is the genetic ancestry at a particular chromosomal location. Local ancestry inference in HCHS/SOL was performed as previously described.^37^ In brief, local African, Amerindian, and European ancestries were inferred from a set of quality-controlled SNPs across the genome using RFMix^38^ and were used to calculate the average values of local ancestries at 14,815 nonoverlapping intervals (local ancestry intervals [LAIs]) on autosomal chromosomes, each spanning tens to hundreds of thousands of base pairs. At each LAI, an individual can carry 0, 1, or 2 copies (counts) of an allele derived from each ancestral population.

We tested the association of WMHs with LAI counts of African, Amerindian, and European ancestries individually and, in secondary analyses, of all ancestries jointly. We used the same linear models as described above and implemented in GENESIS.^31^ Based on previously reported simulation analyses in HCHS/SOL, a *p* value threshold of 5.7 × 10^−5^ controls the family-wise error rate of admixture mapping at level 0.05 and was chosen as the significance threshold.^37^

To prioritize genetic variants underlying the admixture signal, we examined SNP associations within the WMH-associated LAI as previously described.^39^ In addition to the main effects of SNPs, we also investigated SNP-by-LAI count interaction effects. Conditional admixture analysis was then performed including the candidate SNPs as covariate in the admixture mapping model described above.

### Polygenic Scores for WMHs

We evaluated the association of WMHs with polygenic scores (PGSs) constructed from multiple approaches:

1. Weighted Genetic Risk Score: We first used the 27 known WMH SNPs to construct a weighted risk score summing across the WMH risk alleles, with weights taken from the published WMH GWASs.^13^
2. PRSice-2^40^: We applied a clumping and association (C + T) method implemented in PRSice v.2.3.5, using default settings and summary statistics from the published WMH GWASs in NHW individuals.^13^ The best-fit PGS was selected at the *p* value threshold where the model fit had the highest R^2^ score.
3. LDPred2^41^: We applied LDPred2, a new version of LDpred, which derives PGSs based on summary statistics and a LD matrix, following the developer's guide.^42^ We used the ‘auto’ option that directly estimates the 2 LDpred parameters from data. The summary statistics were from the published WMH GWASs in NHW individuals^13^, and the LD matrix (correlations between pairs of genetic variants) was derived from 1,444,196 HapMap3+ variants based on European individuals of the UK Biobank.^43^
4. PRS-CSx^44^: We applied PRS-CSx, a Bayesian polygenic modeling method that leverages population-specific LD and shared genetic information between populations through joint modeling of multiple GWAS summary data.^44^ We used the summary statistics from the WMH GWASs in NHW and AA populations.^13^ We used precomputed LD reference panels constructed using the 1000 Genomes project, which matched the ancestry of each of the GWAS summary statistics. NHW and AA posterior effect size estimates were combined using an inverse variance–weighted meta-analysis within the Gibbs sampler (via the “--meta” option provided by software). From these data, 3 PGSs were constructed, which are based on the meta-analysis weights (PGS-CSx_meta) and the weights derived from the NHW and AA populations only (PGS_CSx_NHW, PGS_CSx_AA).

All derived PGSs were standardized within the genetic ancestry group and evaluated in association analyses using generalized linear mixed models implemented in the GENESIS package as described above. All analyses were adjusted for age at MRI, sex, total intracranial volume, MRI scanner, and 5 principal components of ancestry.

### Data Availability

The main summary statistics that support the findings of this study are available within the Supplementary Data. All primary data used in this study are available from HCHS/SOL. Restrictions apply to the availability of these data, which were used under license for this study. Data are available from sites.cscc.unc.edu/hchs with the permission of HCHS/SOL. Full GWAS summary statistics are available from the authors on request.

## Results

Characteristics of the study sample are summarized in Table 1. The mean age of the participants was 62 years, and 66% were female.

**Table 1:** Characteristics of the Study Sample

	Total sample	Mainland group	Caribbean group
Sample size	2,159	1,234	925
Number of women (%)	1,424 (66.0)	834 (67.6)	590 (63.8)
Mean age (SD), y	62.4 (9.3)	62.4 (9.2)	62.4 (9.4)
Mean log-WMHs (SD)	−0.17 (1.59)	−0.51 (1.52)	0.28 (1.57)

Abbreviation: WMHs = white matter hyperintensities.

### Estimated WMH SNP-Based Heritability and Transferability of Known GWAS Loci in Hispanic/Latino Adults

The estimated SNP-based heritability (h^2^) of WMH volume (inverse normal transformed) was 29% (95% CI 2%–59%) in the total sample, the same estimate as previously reported in NHW individuals.^13^ A similar estimate was obtained for log-transformed WMHs (h^2^ = 28%, 95% CI 1%–58%). Excluding close relatives (N = 59) reduced SNP-based heritability estimates to 13% (95% CI 0%–46%) for both traits. Given the wide 95% CIs, a larger sample will be needed to obtain a more accurate estimate of SNP-based heritability in Hispanic/Latino adults.

We examined whether 27 genetic loci previously identified in the largest published GWAS of WMHs in 50,970 middle-aged and older adults (95% NHW, 5% AA) were reproducible in Hispanic/Latino adults. To account for differences in LD, we evaluated transferability based on credible sets of variants per locus rather than lead variants alone. In our diverse sample of Hispanic/Latino adults, we observed evidence of significant SNP association (*p* < 0.05) at 15 loci and suggestive association (*p* < 0.10) at 5 additional loci (Table 2). The strongest association was observed for rs7596872 at 2p16.1, annotated as an expression quantitative trait locus (eQTL) of *EFEMP1* and the lead SNP in the reported GWAS. With few exceptions, the strongest associations observed in this diverse sample of Hispanic/Latino adults were not with the reported GWAS SNP. Because low statistical power may hinder our ability to make appropriate conclusions regarding a locus' transferability, we calculated the statistical power to observe an association of each of the known WMH loci in our Hispanic/Latino population. Not unexpectedly, power to detect an association was generally low across the loci, except for 17q25.1 and 10p14, the strongest associations reported to date in NHW and AA populations, respectively. While evidence of transferability was observed for 17q25.1, this was not the case for 10p14. Of interest, at 10p14, there was no association of any SNPs with WMHs in the total sample or the Mainland group. By contrast, there was suggestive evidence of an association in the Caribbean group (*p* = 0.08) that has a higher proportion of African ancestry.

**Table 2:** Transferability of WMH Loci in Hispanic/Latino Adults†

Locus	nCS	Min_*p* value			Min_Q value			Power^a^	Top_SNP	Lead SNP?	Implicated gene(s) via eQTL (tissue)
		ALL	ML	CB	All	ML	CB				
1p22.2_PKN2	24	**0.017**	**0.011**	0.325	0.134	0.115	0.743	0.19	rs10922489	No	*KYAT3* (brain)
1q41_KCNK2	3	0.478	*0.056*	0.120	0.975	0.214	0.242	0.15			
2p16.1_EFEMP1	8	**0.001**	**0.016**	**0.011**	0.012	0.128	0.091	0.47	rs7596872	Yes	*EFEMP1* (brain)
2p21_HAAO	12	0.406	0.482	0.228	0.934	0.985	0.913	0.25			
2q32.1_CALCRL	2	0.405	0.068	0.543	0.447	0.088	0.545	0.16			
2q33.2_CARF	99	0.161	0.405	*0.073*	0.412	0.901	0.375	0.26			
3q27.1_KLHL24	160	0.213	0.458	0.103	0.613	0.802	0.495	0.31			
5q14.2_VCAN	8	**0.041**	**0.021**	0.282	0.191	0.103	0.941	0.26	rs10052710	No	*VCAN* (blood)
5q23.2_LOC100505841	23	*0.095*	0.149	**0.039**	0.553	0.974	0.646	0.31	rs17148941	No	*SNCAIP* (brain, heart)
6q25.1_PLEKHG1	23	**0.006**	**0.012**	0.287	0.139	0.160	0.998	0.34	rs9383542	No	*PLEKHG1* (blood)
8p23.1_XKR6	42	**0.039**	**0.017**	0.302	0.402	0.081	0.993	0.17	rs17783634	No	*FAM167A* (blood); *XKR6, SLC35G5* (blood, brain)
8p23.1_PRAG1	8	*0.070*	0.556	**0.047**	0.171	0.831	0.247	0.16	rs17149723	No	*FAM85B* (brain); *ALG1L13P*, *MFHAS1* (blood)
8p23.1_TNKS	75	0.243	0.117	0.240	0.663	0.464	0.999	0.20			
10p14_ECHDC3	16	0.597	0.117	*0.079*	0.926	0.172	0.167	0.99	rs10752232, rs10795889	No	*ECHDC3* (blood)
10q24.33_SH3PXD2A	3	**0.011**	*0.063*	0.101	0.032	0.189	0.195	0.29	rs879655		*SH3PXD2A, ATP5MK* (blood)
10q24.33_SH3PXD2A-AS1	30	0.151	0.186	0.151	0.631	0.538	0.989	0.22			
10q24.33_SH3PXD2A	24	0.181	0.146	0.244	0.932	0.515	0.655	0.28			
13q34_COL4A2	6	0.778	0.783	0.616	0.858	0.195	0.937	0.17			
14q22.1_NID2	22	0.139	0.770	0.124	0.520	0.997	0.628	0.16			
14q32.11_CCDC88C	47	**0.004**	0.127	**0.005**	0.101	0.725	0.050	0.22	rs8021811	No	*CCDC88C* (brain, blood)
14q32.2_DEGS2	18	**0.016**	**0.006**	0.329	0.117	0.020	0.967	0.22	rs8016001	No	*DEGS2* (brain); *WARS1, SLC25A29* (blood)
15q22.31_RASL12	19	**0.038**	**0.016**	0.699	0.171	0.075	0.997	0.16	rs7170256	No	*RASL12* (brain); *ANKDD1A* (blood)
16q12.1_SALL1	5	*0.088*	0.270	0.240	0.136	0.356	0.314	0.22			
16q24.2_C16orf95	47	**0.009**	0.108	**0.013**	0.103	0.488	0.134	0.37	rs4843552	No	*AC136285.1* (CNS)
17q21.31_NMT1	58	**0.005**	**0.044**	**0.030**	0.022	0.184	0.132	0.37	rs6503419	No	*DCAKD, NMT1* (brain, blood, others)
17q25.1_TRIM65	48	**0.005**	0.144	**0.024**	0.069	0.568	0.211	0.82	rs3744027	No	*TRIM65*, *TRIM47* (brain, blood, others)
22q12.1_MN1	16	**0.029**	*0.089*	0.135	0.114	0.200	0.344	0.25	rs5762197	Yes	

Abbreviations: ALL = total sample; CB = Caribbean group; eQTL = expression quantitative trait locus; Lead SNP? = top SNP is the reported GWAS SNP; Min_P/Q = minimum P/Q value for SNPs in the credible set; ML = Mainland group; n_CS = number of SNPs in the credible set; Top_SNP = SNP with the strongest association in the HCHS/SOL Hispanic/Latino group.

a Power calculated in the total sample.

† Values in bold indicate significant evidence of locus transferability. Values in Italic indicates suggestive evidence of transferability.

To investigate the extent of sharing of causal variants between ancestries at the known WMH GWAS loci, we applied a trans-ancestry colocalization method,^35^ which estimates the likelihood of sharing a causal variant accounting for LD. We found significant evidence of sharing at 14q32.11 (*p* = 0.01) and 5q14.2 (*p* = 0.02), as well as suggestive evidence at 17q21.31 (*p* = 0.05).

### PGSs for WMHs in Hispanic/Latino Adults

To further evaluate the relevance of WMH genetic associations from NHW and AA populations in our Hispanic/Latino cohort, we constructed PGSs using 4 methods and tested their association with WMHs. Except for the PGSs constructed from the WMH GWAS in AA populations, all PGSs were associated with WMHs. The strongest association was with the LDPred2-derived PGS, which explained 2.8% of the variance in WMHs (Table 3).

**Table 3:** Association of Polygenic Scores With WMHs in Hispanic/Latino Adults

Score	Beta	SE	*p* Value	% variance explained
wGRS_27SNPs	0.158	0.028	<0.0001	1.05
PGS_PRSice2	0.225	0.028	<0.0001	2.07
PGS_LDPred2	0.266	0.028	<0.0001	2.85
PGS_CSx_NHW	0.216	0.029	<0.0001	1.81
PGS_CSx_AA	0.014	0.029	0.64	0.01
PGS_CSx_meta	0.056	0.028	0.049	0.13

Abbreviation: SE = standard error.

Models adjusted for age, sex, Latino background, total intracranial volume, and MRI scanner.

### Genome-Wide Association Analysis and Meta-Analyses

The Manhattan plot and QQ plot for the GWAS of WMHs in our sample of diverse Hispanic/Latino adults are shown in eFigure 1. There was no evidence of genomic inflation (λ = 1.0). No SNP association reached genome-wide significance. However, 8 loci were suggestively associated with WMHs (*p* < 1 × 10^−6^) (eTable 1). Associated SNPs in these loci were located in intronic and intergenic regions (eFigure 2). The strongest association was with a locus on 6q15 located in an intergenic region near *MAP3K7* and tagged by rs2325337 (*p* = 2.7 × 10^−7^). These SNPs were either not present or not associated with WMHs in the published GWAS in NHW or AA populations (eTable 1).

Gene-based analyses, likewise, identified 4 suggestive associations (*p* < 2.6 × 10^−5^) (eTable 2). The strongest association was with *PPP2R3C* (*p* = 7.7 × 10^−6^). Although there was some variation in the ranking of *p* values, there was almost complete overlap of the loci identified in GWAS results of log-transformed and inverse normal–transformed WMHs (eTables 3 and 4).

We meta-analyzed our GWAS results with those previously published,^13^ which include a set of summary results from NHW populations and 1 from AA populations. A total of 18 loci reached genome-wide significance, including 1 novel locus on chromosome 12q22 tagged by rs10859915 (*p* = 2.6 × 10^−8^) (eTable 5 and eFigure 3). Functional annotation suggests that this variant is located in a region of open chromatin and is a strong eQTL of *AMDHD1* (eFigure 4).

### Admixture Mapping Analyses

**Figure 1:**
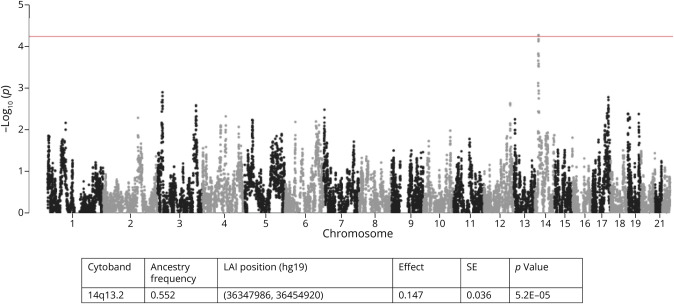
Association of WMH Volume With European Local Ancestry The red line represents the threshold for genome-wide statistical significance. Information about the associated LAI is given in the table. LAI = local ancestry interval.

In admixture mapping analyses, we identified a statistically significant local ancestry–associated region for WMHs on 14q13.2 (Figure 1), where higher counts of European ancestry were associated with a higher WMH volume (*p* = 5.3 × 10^−7^). There was no association of counts of African or Amerindian ancestry with WMHs. In the 14q13.2 region, no variants showed association with WMHs in the large published GWAS of NHW adults (eFigure 5), casting doubts about the role of common variants in explaining the admixture peak. Of interest, this region is immediately adjacent to the suggestive locus identified in our GWAS and tagged by rs555928282 (minor allele frequency (MAF = 0.14)). This SNP is rare in populations of European ancestry (MAF = 0.005) and thus was not present in the published GWAS. Functional annotation of this variant indicates that it is a protein QTL for *FAM177A1* (eTable 1). Association analyses conditioning on this SNP, however, only partially abrogated the admixture signal (*p* = 6.9 × 10^−4^). Modeling the interaction of SNPs with the count of European ancestry in the region of the admixture signal identified rs10467758 (MAF = 0.011), with the strongest interaction effect on WMHs (beta = −1.11; *p* = 2.6 × 10^−4^) (eFigure 6). There was no association of the main effects of that SNP with WMHs. This SNP is located in an intron of *BRMS1L* and is very rare in populations of European ancestry (MAF< 0.001) but more common in populations of African ancestry (MAF = 0.06). Association analyses conditioning on this interaction did not fully abrogate the admixture signal (*p* = 1.8 × 10^−4^) while conditioning on both rs555928282 (main effect) and rs10467758 (interaction effect) further dampened the admixture signal (*p* = 1.9 × 10^−3^). Taken together, these results are consistent with the hypothesis that a rare haplotype, not well tagged by any single GWAS variant, underlies the observed admixture signal. To further investigate this hypothesis, we examined the association of genes mapping to chr14q13.2 and aggregating the effects of putatively functional low-frequency and rare coding variants in 20,719 stroke/dementia-free adults from the Cohorts for Heart and Aging Research in Genomic Epidemiology consortium.^12^ We identified an association of rare coding variants in *RALGAPA1* with WMHs (*p* = 0.018) (eTable 6).

A summary of the identified genetic loci associated with WMHs in Hispanic/Latino individuals is provided in eTable 7.

## Discussion

We investigated the genetic architecture of WMHs in a large sample of diverse Hispanic/Latino adults. We found that multiple genetic loci previously identified in populations of European and African ancestry are transferable to a diverse Hispanic/Latino population and identified loci with evidence of shared causal variants. Finally, using GWAS meta-analysis and admixture mapping approaches, we identified novel loci associated with WMHs, contributing new knowledge about this important marker of brain vascular injury.

To account for differences in LD structure among populations, we assessed the transferability of known WMH loci based on credible sets of variants rather than the locus sentinel SNP alone. A total of 20 loci showed some evidence of transferability in Hispanic/Latino individuals. Except for 2p16.1 and 22q12.1, the SNP with the strongest association in Hispanic/Latino individuals differed from the sentinel SNP in the published GWAS, underscoring the potential of diverse populations for fine-mapping. Indeed, the SNPs identified in our transferability analyses were functionally annotated as eQTLs, thereby implicating several candidate genes. Moreover, there was evidence of sharing of causal variants at 14q32.11, 5q14.2, and 17q21.31. The strongest associations at these 3 loci in our sample were observed for rs8021811, rs10052710, and rs6503419, respectively. Functional annotation of these variants implicated *CCDC88C, VCAN*, *DCAKD*, and *NMT1* as candidate genes in WMH burden. *CCDC88C* encodes a coiled-coil domain-containing protein that regulates the Wnt signaling pathway. Rare pathogenic variants in this gene have been implicated in congenital hydrocephalus and spinocerebellar ataxia.^45-47^ *VCAN* encodes versican, a large chondroitin sulfate proteoglycan and a major component of the extracellular matrix. *DCAKD* encodes the dephospho-CoA kinase domain-containing protein, with a putative role in synaptic development.^48^ *NMT1* encodes N-myristoyltransferase 1, an enzyme involved in the lipid modification of certain cellular and viral proteins, which has been implicated in several cancers and HIV infection.^49^ Expression of *DCAKD* and *NMT1* has been associated with white matter microstructure in the UK Biobank.^50^ Our study lacked power to conclusively assess transferability at several of the known GWAS loci. One notable exception is the locus on 10p14, previously identified in a population of African ancestry. Despite sufficient power, we did not detect an association of this locus with WMHs in our total sample. However, there was a suggestive association in the Caribbean group, which has a higher proportion of African ancestry. These results illustrate the challenge in addressing transferability of genetic loci in the Hispanic/Latino population where a population label based on continental ancestry may not adequately reflect the diversity and continuum of genetic ancestries.

Addressing this challenge will require increasing Hispanic/Latino representation in genetic studies of WMHs. To date, only 1 GWAS of WMHs has been conducted in 922 Caribbean Hispanic older adults, which did not identify any significant associations.^18^ Although our GWAS included the largest and most diverse sample of Hispanic/Latino individuals to date with WMH and genetic data, we did not identify any SNP or gene-based association at genome-wide significance level. However, meta-analysis of our results with those of the previously published GWAS identified a novel locus at 12q22 tagged by rs10859915, an intergenic variant located downstream of *NTN4* and annotated as an eQTL of *AMDHD1*. *NTN4* encodes netrin 4, a laminin-like secreted protein regulating axonal guidance and angiogenesis.^51^ Human genetic evidence provided very strong support that this gene influences diastolic blood pressure, and indeed, rs10859915 showed associations with systolic and diastolic blood pressure, both major risk factors of WMHs.^52^ *AMDHD1* encodes amidohydrolase domain-containing protein 1, an enzyme involved in the metabolism of histidine, phenylalanine, tyrosine, tryptophan, and vitamin D. Recent research suggests a role of *AMDHD1* in the activation of TGF-β signaling pathway.^53^ Altered TGF-β has been implicated in the pathogenesis of cerebral small vessel disease and vascular contributions to cognitive impairment and dementia in humans and rodent models.^54-56^

Exploiting admixture patterns within large chromosomal intervals pinpointed a novel locus on chr14q13.2 associated with WMHs. Because admixture mapping captures the effect of genetic variation with a wider allele frequency spectrum than GWAS, it is a powerful complementary method to GWAS. To date, few studies have examined the association of rare variants with WMHs.^12,57^ Several lines of evidence converge to suggest an association of rare variants at chr14q13.2 with WMHs. First, the admixture peak lies in the vicinity of the region identified in our GWAS and tagged by a rare variant in European ancestry population. Functional annotation of this variant and gene-based analyses implicate *FAM177A1*. This gene encodes a Golgi complex–localized protein with function in the innate immune response as a negative regulator of the IL-1β and NF-κB inflammatory cascade.^58,59^ Rare biallelic loss-of-function variants in *FAM177A1* cause a neurodevelopmental disorder featuring brain MRI abnormalities in the white matter and gait disturbance.^60^ Second, the SNP identified in SNP-by-local ancestry interaction is extremely rare in NHW individuals while more common in AA individuals, further supporting the hypothesis of rare variation in this region influencing WMH volume. Finally, association analyses of aggregated rare coding variants conducted in a large sample of NHW individuals identified *RALGAPA1*, a gene highly expressed in the brain and encoding the catalytic subunit of Ral GTPase–activating protein. Adult mice deficient for Ral GTPases in oligodendrocytes exhibit myelination defects and degeneration of the myelin-axon unit.^61^ Rare loss-of-function variants in this gene have been associated with severe neurodevelopmental disorders, with dysplasia and thinning of the corpus callosum.^62^ Of interest, *RALGAPA1* was brought forth in gene-based association analyses from a large GWAS of stroke in a population of European ancestry, although single-variant analyses did not identify this locus.^63^

We observed a strong association of PGSs developed from predominantly European ancestry GWAS results with WMHs in our sample of diverse Hispanic/Latino adults. Incorporating summary statistics from African ancestry GWAS data using PRS-CSx^44^ did not strengthen association of the polygenic score in our sample. This is likely due to the limited sample size of the African ancestry GWAS. Notably, if causal variants are shared across ancestries, much can be gained from large European ancestry GWASs.

Our study has several limitations: First, our sample size was limited and did not afford sufficient power to uncover novel GWAS loci in Hispanic/Latino adults. Second, given the challenge in establishing appropriate statistical significance thresholds for loci with prior evidence of WMH association, we used the usual *p* = 0.05 threshold in our transferability analyses, which may not sufficiently account for the multiple tests performed. Third, meta-analyses with the largest WMH GWAS data identified a novel locus that needs to be independently confirmed. Finally, while we provided support for the role of rare variation at chr14q13.2, possibly involving *RALGAPA1,* we have not directly examined rare variants in our study. Results such as these underscore the need for additional studies investigating the role of rare variants in the genetic architecture of WMHs.

In conclusion, our study provides an assessment of the transferability of WMH loci to Hispanic/Latino adults, with implications for fine-mapping and risk prediction. It also uncovers novel loci harboring candidate genes with strong biological relevance to WMH etiology and pathophysiology.

## Glossary

AA: African American
eQTL: expression quantitative trait locus
FLAIR: fluid-attenuated inversion recovery
GENESIS: GENetic EStimation and Inference in Structured sample
GWAS: genome-wide association study
LAI: local ancestry interval
LD: linkage disequilibrium
NHW: non-Hispanic White
PGS: polygenic score
SNP: single-nucleotide polymorphism
WHM: white matter hyperintensity

## References

[1] Wardlaw JM, Smith EE, Biessels GJ, et al. Neuroimaging standards for research into small vessel disease and its contribution to ageing and neurodegeneration. Lancet Neurol. 2013;12(8):822-838. doi:10.1016/S1474-4422(13)70124-8 23867200 PMC3714437

[2] Wardlaw JM, Smith C, Dichgans M. Small vessel disease: mechanisms and clinical implications. Lancet Neurol. 2019;18(7):684-696. doi:10.1016/S1474-4422(19)30079-1 31097385

[3] Debette S, Seshadri S, Beiser A, et al. Midlife vascular risk factor exposure accelerates structural brain aging and cognitive decline. Neurology 2011;77(5):461-468. doi:10.1212/WNL.0b013e318227b227 21810696 PMC3146307

[4] Maillard P, Seshadri S, Beiser A, et al. Effects of systolic blood pressure on white-matter integrity in young adults in the Framingham Heart Study: a cross-sectional study. Lancet Neurol. 2012;11(12):1039-1047. doi:10.1016/S1474-4422(12)70241-7 23122892 PMC3510663

[5] Debette S, Beiser A, DeCarli C, et al. Association of MRI markers of vascular brain injury with incident stroke, mild cognitive impairment, dementia, and mortality: the Framingham Offspring Study. Stroke. 2010;41(4):600-606. doi:10.1161/STROKEAHA.109.570044 20167919 PMC2847685

[6] Liao D, Cooper L, Cai J, et al. Presence and severity of cerebral white matter lesions and hypertension, its treatment, and its control. The ARIC Study. Atherosclerosis Risk in Communities Study. Stroke. 1996;27(12):2262-2270. doi:10.1161/01.str.27.12.2262 8969791

[7] Atwood LD, Wolf PA, Heard-Costa NL, et al. Genetic variation in white matter hyperintensity volume in the Framingham Study. Stroke. 2004;35(7):1609--1613. doi:10.1161/01.STR.0000129643.77045.10 15143299

[8] Carmelli D, DeCarli C, Swan GE, et al. Evidence for genetic variance in white matter hyperintensity volume in normal elderly male twins. Stroke. 1998;29(6):1177-1181. doi:10.1161/01.str.29.6.1177 9626291

[9] Turner ST, Jack CR, Fornage M, Mosley TH, Boerwinkle E, de Andrade M. Heritability of leukoaraiosis in hypertensive sibships. Hypertension. 2004;43(2):483-487. doi:10.1161/01.HYP.0000112303.26158.92 14718359

[10] Fornage M, Debette S, Bis JC, et al. Genome-wide association studies of cerebral white matter lesion burden: the CHARGE consortium. Ann Neurol. 2011;69(6):928-939. doi:10.1002/ana.22403 21681796 PMC3122147

[11] Verhaaren BF, Debette S, Bis JC, et al. Multiethnic genome-wide association study of cerebral white matter hyperintensities on MRI. Circ Cardiovasc Genet. 2015;8(2):398-409. doi:10.1161/CIRCGENETICS.114.000858 25663218 PMC4427240

[12] Jian X, Satizabal CL, Smith AV, et al. Exome chip analysis identifies low-frequency and rare variants in MRPL38 for white matter hyperintensities on brain magnetic resonance imaging. Stroke. 2018;49(8):1812-1819. doi:10.1161/STROKEAHA.118.020689 30002152 PMC6202149

[13] Sargurupremraj M, Suzuki H, Jian X, et al. Cerebral small vessel disease genomics and its implications across the lifespan. Nat Commun. 2020;11(1):6285. doi:10.1038/s41467-020-19111-2 33293549 PMC7722866

[14] Sirugo G, Williams SM, Tishkoff SA. The missing diversity in human genetic studies. Cell. 2019;177(4):1080-1131. doi:10.1016/j.cell.2019.04.032 31051100

[15] Martin AR, Kanai M, Kamatani Y, Okada Y, Neale BM, Daly MJ. Clinical use of current polygenic risk scores may exacerbate health disparities. Nat Genet. 2019;51(4):584-591. doi:10.1038/s41588-019-0379-x 30926966 PMC6563838

[16] Census Bureau Facts for Features. U.S. Census Bureau. 2024. Accessed September 15, 2025. https://www.census.gov/newsroom/facts-for-features/2024/hispanic-heritage-month.html

[17] Farkhondeh V, DeCarli C. White matter hyperintensities in diverse populations: a systematic review of literature in the United States. Cereb Circ Cogn Behav. 2024;6:100204. doi:10.1016/j.cccb.2024.100204 38298455 PMC10828602

[18] Beecham A, Dong C, Wright CB, et al. Genome-wide scan in Hispanics highlights candidate loci for brain white matter hyperintensities. Neurol Genet 2017;3(5):e185. doi:10.1212/NXG.0000000000000185 28975155 PMC5619914

[19] Lavange LM, Kalsbeek WD, Sorlie PD, et al. Sample design and cohort selection in the hispanic community health study/study of Latinos. Ann Epidemiol. 2010;20(8):642-649. doi:10.1016/j.annepidem.2010.05.006 20609344 PMC2921622

[20] Sorlie PD, Aviles-Santa LM, Wassertheil-Smoller S, et al. Design and implementation of the hispanic community health study/study of Latinos. Ann Epidemiol. 2010;20(8):629-641. doi:10.1016/j.annepidem.2010.03.015 20609343 PMC2904957

[21] Gonzalez HM, Tarraf W, Fornage M, et al. A research framework for cognitive aging and Alzheimer's disease among diverse US Latinos: design and implementation of the hispanic community health study/study of latinos-investigation of neurocognitive aging (SOL-INCA). Alzheimers Dement. 2019;15(12):1624-1632. doi:10.1016/j.jalz.2019.08.192 31759880 PMC6925624

[22] Fletcher E, DeCarli C, Fan AP, Knaack A. Convolutional neural net learning can achieve production-level brain segmentation in structural magnetic resonance imaging. Front Neurosci. 2021;15:683426. doi:10.3389/fnins.2021.683426 34234642 PMC8255694

[23] DeCarli C, Miller BL, Swan GE, et al. Predictors of brain morphology for the men of the NHLBI twin study. Stroke. 1999;30(3):529-536. doi:10.1161/01.str.30.3.529 10066847

[24] Maillard P, Lu H, Arfanakis K, et al. Instrumental validation of free water, peak-width of skeletonized mean diffusivity, and white matter hyperintensities: MarkVCID neuroimaging kits. Alzheimers Dement (Amst). 2022;14(1):e12261. doi:10.1002/dad2.12261 35382232 PMC8959640

[25] Stickel AM, Tarraf W, Gonzalez KA, et al. Characterizing age- and sex-related differences in brain structure among middle-aged and older Hispanic/Latino adults in the study of Latinos- investigation of neurocognitive aging magnetic resonance imaging (SOL-INCA MRI). Neurobiol Aging. 2023;126:58-66. doi:10.1016/j.neurobiolaging.2023.02.007 36933278 PMC10363333

[26] Conomos MP, Laurie CA, Stilp AM, et al. Genetic diversity and association studies in US hispanic/latino populations: applications in the hispanic community health study/study of Latinos. Am J Hum Genet. 2016;98(1):165-184. doi:10.1016/j.ajhg.2015.12.001 26748518 PMC4716704

[27] Laurie CC, Doheny KF, Mirel DB, et al. Quality control and quality assurance in genotypic data for genome-wide association studies. Genet Epidemiol. 2010;34(6):591-602. doi:doi:10.1002/gepi.20516 20718045 PMC3061487

[28] Kowalski MH, Qian H, Hou Z, et al. Use of >100,000 NHLBI Trans-Omics for Precision Medicine (TOPMed) Consortium whole genome sequences improves imputation quality and detection of rare variant associations in admixed African and Hispanic/Latino populations. Plos Genet. 2019;15(12):e1008500. doi:10.1371/journal.pgen.1008500 31869403 PMC6953885

[29] Chen GB. Estimating heritability of complex traits from genome-wide association studies using IBS-based Haseman-Elston regression. Front Genet. 2014;5:107. doi:10.3389/fgene.2014.00107 24817879 PMC4012219

[30] Sofer T. Confidence intervals for heritability via Haseman-Elston regression. Stat Appl Genet Mol Biol. 2017;16(4):259-273. doi:10.1515/sagmb-2016-0076 28862991 PMC5857391

[31] Gogarten SM, Sofer T, Chen H, et al. Genetic association testing using the GENESIS R/Bioconductor package. Bioinformatics. 2019;35(24):5346-5348. doi:10.1093/bioinformatics/btz567 31329242 PMC7904076

[32] de Leeuw CA, Mooij JM, Heskes T, Posthuma D. MAGMA: generalized gene-set analysis of GWAS data. Plos Comput Biol. 2015;11(4):e1004219. doi:10.1371/journal.pcbi.1004219 25885710 PMC4401657

[33] Willer CJ, Li Y, Abecasis GR. METAL: fast and efficient meta-analysis of genomewide association scans. Bioinformatics. 2010;26(17):2190-2191. doi:10.1093/bioinformatics/btq340 20616382 PMC2922887

[34] Magi R, Horikoshi M, Sofer T, et al. Trans-ethnic meta-regression of genome-wide association studies accounting for ancestry increases power for discovery and improves fine-mapping resolution. Hum Mol Genet. 2017;26(18):3639-3650. doi:doi:10.1093/hmg/ddx280 28911207 PMC5755684

[35] Kuchenbaecker K, Telkar N, Reiker T, et al. The transferability of lipid loci across African, Asian and European cohorts. Nat Commun. 2019;10(1):4330. doi:10.1038/s41467-019-12026-7 31551420 PMC6760173

[36] Chun S, Casparino A, Patsopoulos NA, et al. Limited statistical evidence for shared genetic effects of eQTLs and autoimmune-disease-associated loci in three major immune-cell types. Nat Genet. 2017;49(4):600-605. doi:10.1038/ng.3795 28218759 PMC5374036

[37] Browning SR, Grinde K, Plantinga A, et al. Local ancestry inference in a large US-based hispanic/latino study: hispanic community health study/study of Latinos (HCHS/SOL). G3 (Bethesda). 2016;6:1525-1534. doi:10.1534/g3.116.028779 27172203 PMC4889649

[38] Maples BK, Gravel S, Kenny EE, Bustamante CD. RFMix: a discriminative modeling approach for rapid and robust local-ancestry inference. Am J Hum Genet. 2013;93(2):278-288. doi:10.1016/j.ajhg.2013.06.020 23910464 PMC3738819

[39] Xia R, Jian X, Rodrigue AL, et al. Admixture mapping of cognitive function in diverse hispanic and Latino adults: results from the hispanic community health study/study of Latinos. Alzheimers Dement. 2024;20(9):6070-6081. doi:10.1002/alz.14082 38946675 PMC11497725

[40] Choi SW, O'Reilly PF. PRSice-2: polygenic Risk Score software for biobank-scale data. Gigascience. 2019;8(7):giz082. doi:10.1093/gigascience/giz082 31307061 PMC6629542

[41] Prive F, Arbel J, Vilhjalmsson BJ. LDpred2: better, faster, stronger. Bioinformatics. 2021;36(22-23):5424-5431. doi:10.1093/bioinformatics/btaa1029 33326037 PMC8016455

[42] Prive F. Polygenic scores and inference using LDpred2. Accessed March 15, 2024. privefl.github.io/bigsnpr/articles/LDpred2.html

[43] Prive F, Albinana C, Arbel J, Pasaniuc B, Vilhjalmsson BJ. Inferring disease architecture and predictive ability with LDpred2-auto. Am J Hum Genet 2023;110(12):2042-2055. doi:10.1016/j.ajhg.2023.10.010 37944514 PMC10716363

[44] Ruan Y, Lin YF, Feng YCA, et al. Improving polygenic prediction in ancestrally diverse populations. Nat Genet. 2022;54(5):573-580. doi:10.1038/s41588-022-01054-7 35513724 PMC9117455

[45] Tsoi H, Yu AC, Chen ZS, et al. A novel missense mutation in CCDC88C activates the JNK pathway and causes a dominant form of spinocerebellar ataxia. J Med Genet. 2014;51(9):590-595. doi:10.1136/jmedgenet-2014-102333 25062847 PMC4145425

[46] Drielsma A, Jalas C, Simonis N, et al. Two novel CCDC88C mutations confirm the role of DAPLE in autosomal recessive congenital hydrocephalus. J Med Genet. 2012;49(11):708-712. doi:10.1136/jmedgenet-2012-101190 23042809

[47] Ekici AB, Hilfinger D, Jatzwauk M, et al. Disturbed Wnt signalling due to a mutation in CCDC88C causes an autosomal recessive non-syndromic hydrocephalus with medial diverticulum. Mol Syndromol. 2010;1(3):99-112. doi:10.1159/000319859 21031079 PMC2957845

[48] Gonzalez-Lozano MA, Klemmer P, Gebuis T, et al. Dynamics of the mouse brain cortical synaptic proteome during postnatal brain development. Sci Rep 2016;6:35456. doi:10.1038/srep35456 27748445 PMC5066275

[49] Selvakumar P, Kumar S, Dimmock JR, Sharma RK. NMT1 (N- 1). Atlas Genet Cytogenet Oncol Haematol. 2011;15(7):570-575. doi:10.4267/2042/45997 22977462 PMC3439497

[50] Barbu MC, Spiliopoulou A, Colombo M, et al. Expression quantitative trait loci-derived scores and white matter microstructure in UK Biobank: a novel approach to integrating genetics and neuroimaging. Transl Psychiatry. 2020;10(1):55. doi:10.1038/s41398-020-0724-y 32066731 PMC7026054

[51] Dong F, Liu Y, Yan W, et al. Netrin-4: focus on its role in axon guidance, tissue stability, angiogenesis and tumors. Cell Mol Neurobiol. 2023;43(5):1663-1683. doi:10.1007/s10571-022-01279-4 36350538 PMC11412186

[52] Cerebrovascular Disease Knowledge Portal. Accessed May 21 2025. cerebrovascularportal.org

[53] Ma Z, Sun J, Li Z, Huang S, Li B. AMDHD1 acts as a tumor suppressor and contributes to activation of TGF-β signaling pathway in cholangiocarcinoma. Cell Death Differ. 2025;32(1):162-176. doi:10.1038/s41418-024-01361-y 39143229 PMC11742690

[54] Yamamoto Y, Ihara M. Disruption of transforming growth factor-β superfamily signaling: a shared mechanism underlying hereditary cerebral small vessel disease. Neurochem Int. 2017;107:211-218. doi:10.1016/j.neuint.2016.12.003 28034724

[55] Kandasamy M, Anusuyadevi M, Aigner KM, et al. TGF-beta signaling: a therapeutic target to reinstate regenerative lasticity in vascular dementia? Aging Dis. 2020;11(4):828-850. doi:10.14336/AD.2020.0222 32765949 PMC7390515

[56] Branyan K, Labelle-Dumais C, Wang X, et al. Elevated TGFβ signaling contributes to cerebral small vessel disease in mouse models of Gould syndrome. Matrix Biol. 2023;115:48-70. doi:10.1016/j.matbio.2022.11.007 36435425 PMC10393528

[57] Malik R, Beaufort N, Frerich S, et al. Whole-exome sequencing reveals a role of HTRA1 and EGFL8 in brain white matter hyperintensities. Brain. 2021;144(9):2670-2682. doi:10.1093/brain/awab253 34626176 PMC8557338

[58] Liao BW, Zhang HY, Du WT, Ran Y, Wang YY, Xu ZS. FAM177A1 inhibits IL-1β-induced signaling by impairing TRAF6-Ubc13 association. J Immunol. 2021;207(12):3090-3097. doi:10.4049/jimmunol.2100561 34799425

[59] Chen H, Guo M, Yue D, et al. MicroRNA-7 negatively regulates Toll-like receptor 4 signaling pathway through FAM177A. Immunology. 2021;162(1):44-57. doi:10.1111/imm.13252 32852789 PMC7730018

[60] Kohler JN, Legro NR, Baldridge D, et al. Loss of function of FAM177A1, a Golgi complex localized protein, causes a novel neurodevelopmental disorder. Genet Med. 2024;26(9):101166. doi:10.1016/j.gim.2024.101166 38767059 PMC11451386

[61] DeGeer J, Datwyler AL, Rickenbach C, et al. Ral GTPases are critical regulators of spinal cord myelination and homeostasis. Cell Rep. 2022;40(13):111413. doi:10.1016/j.celrep.2022.111413 36170840

[62] Wagner M, Skorobogatko Y, Pode-Shakked B, et al. Bi-Allelic variants in RALGAPA1 cause profound neurodevelopmental disability, muscular hypotonia, infantile spasms, and feeding abnormalities. Am J Hum Genet. 2020;106(2):246-255. doi:10.1016/j.ajhg.2020.01.002 32004447 PMC7010976

[63] Mishra A, Malik R, Hachiya T, et al. Stroke genetics informs drug discovery and risk prediction across ancestries. Nature. 2022;611(7934):115-123. doi:10.1038/s41586-022-05165-3 36180795 PMC9524349

